# Globus pallidus is not independent from striatal direct pathway neurons: an up-to-date review

**DOI:** 10.1186/s13041-024-01107-4

**Published:** 2024-06-07

**Authors:** Fumino Fujiyama, Fuyuki Karube, Yasuharu Hirai

**Affiliations:** Laboratory of Cytology and Histology, Faculty of Medicine, Hokkaido University, Sapporo, Japan

**Keywords:** Basal ganglia, Striatum, Dopamine

## Abstract

Striatal projection neurons, which are classified into two groups—direct and indirect pathway neurons, play a pivotal role in our understanding of the brain’s functionality. Conventional models propose that these two pathways operate independently and have contrasting functions, akin to an “accelerator” and “brake” in a vehicle. This analogy further elucidates how the depletion of dopamine neurons in Parkinson's disease can result in bradykinesia. However, the question arises: are these direct and indirect pathways truly autonomous? Despite being distinct types of neurons, their interdependence cannot be overlooked. Single-neuron tracing studies employing membrane-targeting signals have shown that the majority of direct pathway neurons terminate not only in the output nuclei, but also in the external segment of the globus pallidus (GP in rodents), a relay nucleus of the indirect pathway. Recent studies have unveiled the existence of arkypallidal neurons, which project solely to the striatum, in addition to prototypic neurons. This raises the question of which type of GP neurons receive these striatal axon collaterals. Our morphological and electrophysiological experiments showed that the striatal direct pathway neurons may affect prototypic neurons via the action of substance P on neurokinin-1 receptors. Conversely, another research group has reported that direct pathway neurons inhibit arkypallidal neurons via GABA. Regardless of the neurotransmitter involved, it can be concluded that the GP is not entirely independent of direct pathway neurons. This review article underscores the intricate interplay between different neuronal pathways and challenges the traditional understanding of their independence.

## Introduction

The striatal projection neurons, integral to our understanding of brain functionality, can be categorized into two distinct groups. The first group corresponds to the direct pathway neurons that express the dopamine D1 receptor (D1R) and substance P (SP); these neurons project directly to the output nuclei, specifically the entopeduncular nucleus (EP)—the homologue of the internal segment of globus pallidus (GPi) in primates—and substantia nigra pars reticulata (SNr). The second group corresponds to the indirect pathway neurons that express the dopamine D2 receptor (D2R) and encephalin. The indirect pathway neurons project to the  globus pallidus (GP)—the homologue of the external segment of globus pallidus (GPe) [1–3].

### Pros and cons of the direct and indirect model

The direct/indirect pathways model was established to explain the physiological and pathological states of behaviors. Both pathways originate from the striatum, activated by the cerebral cortical inputs; however, they affect the downstream nuclei in different, often opposing, manners. It was initially believed that these two types of projection systems function independently and are diametrically opposed in the target regions of the basal ganglia (EP and SNr) similar to a car's accelerator and brake, given the lack of innervation of the relay nucleus of the indirect pathway by the direct pathway neurons [4–7].

Dopamine exerts an excitatory modulation on direct pathway neurons via action at D1R, whereas it affects inhibitory modulation on indirect pathway neurons by actions at D2R [8–10]. Consequently, with the degeneration of dopamine neurons in Parkinson’s disease, the balance of the dual pathways becomes inclined towards braking. This model effectively explains one of the main pathological conditions of Parkinson's disease, i.e., “akinesia” or “hypokinesia,” where the degeneration of dopamine neurons causes a shift towards the “brake.”

The question then arises: how do these two pathways work independently? As striatal projection neurons are not spontaneously active [11, 12], the excitatory inputs arising from the cerebral cortex and/or thalamus are necessary to drive these pathways. It has been hypothesized that differential excitatory innervations onto striatal neurons involved in these two pathways can explain their independence. Anatomical studies conducted on rodents have reported a biased excitatory innervation between the striatal neurons, dependent on the direct/indirect pathways [13, 14]. However, other reports have demonstrated relatively similar inputs and convergence of excitatory inputs onto these two striatal pathway neurons [15, 16]. In vivo recordings from the striatal direct/indirect pathway neurons have conclusively revealed that neurons involved in these two pathways are simultaneously active [17, 18]. Therefore, the behaviors of the direct/indirect pathways may not be as straightforward as initially proposed, prompting a reconsideration of the model or concept. One possible hypothesis is that when the direct pathway neurons, which are responsible for a specific purpose, are active, the indirect pathway neurons, which are not responsible for that purpose, are also active to suppress other behaviors [7, 19]. This has become a hot topic in the field, particularly regarding how such selective circuits can be implemented. According to this concept, the direct/indirect pathways are not independent, because the resulting behaviors are a product of the cooperation of the two pathways.

In this review, we delve into the potential non-independency of the direct/indirect pathways. The original model’s limitation is its inability to account for cooperation between the direct and indirect pathway neurons. When driving a car, we manually control the accelerator and the brake. However, can the direct and indirect neurons, which are distinct neurons, independently control the “accelerator” and “brake” separately to achieve smooth motor control? Recent studies on rodents shed light on the detailed cell types and neuronal connections of the basal ganglia nuclei, not only for the striatum but other nuclei. Although these findings can apply to primates including humans, they have not yet been fully evaluated.

As noted, the imbalance of the direct and indirect pathways, based on a common model of the basal ganglia, elegantly explains many of major symptoms of movement disorders such as bradykinesia, hypokinesia, and akinesia. However, it is challenging to discern the mechanisms of other symptoms, including tremors, rigidity, and the difficulty of voluntary start/stop movements, as well as other non-motor effects. If we could modify the common model using recent advancements in our understanding of the basal ganglia neural circuitries, we may uncover the mechanisms of these yet unexplained symptoms. The rodent studies themselves contain discrepancies, and we are still in the midst of our journey to fully understand these complex systems. Moreover, a significant question remains regarding the extent to which we can apply data from rodent studies to primates, including humans. Recent advancements in RNA-sequencing may shed light on the similarities and differences in the cell types of the basal ganglia among rodents and primates. This is exemplified by one of the Brain Initiative projects, “A cellular atlas of the primate and human basal ganglia” (https://braininitiative.nih.gov/node/3754). However, even if the rodents and primates share the basic cell types, there is no guarantee of similarity in axonal projections for each cell type [20].

In this context, the present narrative review focused on the crosstalk between the direct and indirect pathways via axon collaterals of the direct pathway neurons in the GP. These collaterals exist in both rodents and primates, making them a suitable starting point for discussions on refining the basal ganglia model.

#### Relationship between the two pathway neurons in the striatum

The neostrial projection neurons are composed of two types of medium-sized spiny projection neurons, direct and indirect pathway neurons. Given that the medium spiny projection neurons comprise up to 85% of the striatal neuronal population, the striatum can be conceptualized as a lateral inhibitory network controlled by GABAergic synaptic transmissions between these spiny projection neurons.

Electrophysiological research has shown that the synaptic connections between spiny projection neurons are weaker than previously assumed [21], and this has been elucidated in a review article [22]. Burke et al. used adult rat striatal slices for sharp intracellular electrode recordings and revealed that only 20% of spiny neuron pairs formed unidirectional synapses that were blocked by GABA_A_ antagonists. Moreover, the synaptic responses showed a high failure rate and were small in size, indicating sparse connections [11]. Similarly, studies conducted with young rats and organotypic cultures have shown synaptic connections between only 13% of pairs of spiny neurons, with these connections also displaying high failure rates [23]. Our study using double immunolabelling with the post-embedding immunogold technique also validated that the levels of GABA in presynaptic terminals and GABA receptors at the postsynaptic membranes between projection neurons were less than those at the synapses between the interneurons and projection neurons [24]. Although paired whole-cell recordings between fast-spiking parvalbumin (PV) interneurons and spiny neurons revealed that PV neurons made strong GABAergic connections with spiny neurons (for review article, see [25]), this implies that direct and indirect pathway neurons do not directly affect each other in the striatum. These morphological and electrophysiological findings collectively suggest that the synaptic connection between the spiny neurons is weak.

However, a study by Chuhma et al. [26] reported that spiny neurons in the dorsal striatum form strong connections with other spiny neurons and have weaker connections with tonically active cholinergic interneurons, but not with fast-spiking GABA interneurons. The researchers found that the synaptic connections between spiny neurons, measured in transgenic ChR2 experiments, are significantly greater (63%) than those of paired recordings (10–37%) [11, 26–29]. This discrepancy could be due to the difference between the single and mass inputs or the difference in the extent of recorded regions with the two procedures.

#### Axon collaterals of direct pathway neurons in globus pallidus

**Fig. 1:**
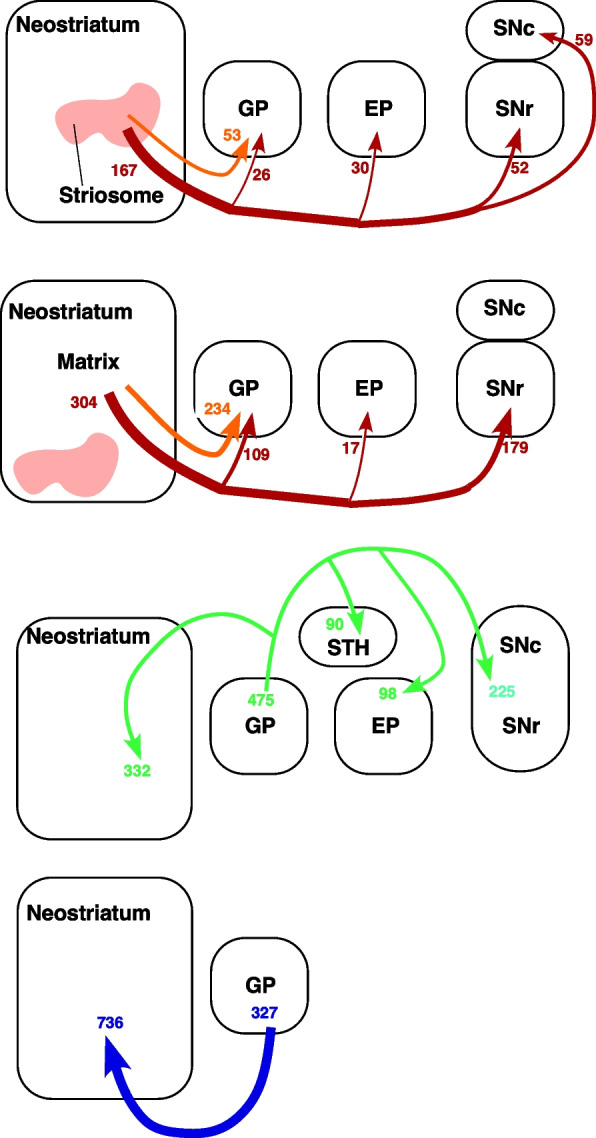
Summary of the single neuron tracing study for striatofugal and pallidofugal neurons Red and orange lines indicate the projections of direct and indirect pathway neurons, respectively, in the striosome (1st raw) and matrix (2nd raw) compartments. Green and blue lines indicate the projections of prototypic and arkypallidal neurons, respectively. Each numeral is the mean number of the axon varicosities (per neuron) that were seen in the corresponding target region. GP, globus pallidus; EP, entopeduncular nucleus; PPT, pedunculopontine tegmental nucleus; SNc, substantia nigra pars compacta; SNr, substantia nigra pars reticulata

The GP is a region that may be implicated in both direct and indirect pathways, apart from the striatum. Earlier reports using biocytin intracellular injection indicated that 5 out of 13 striatal neurons in rats projected axon collaterals to the GP—a relay nucleus of the indirect pathway—and to the EP and SNr [30]; however our previous study, which used a single neuron-tracing method with a membrane-targeting Sindbis virus vector, indicated that the majority of direct pathway neurons in both the striatal striosome and matrix compartments formed arborizations of varicosities in the GP (matrix: 9/10, striosome: 5/8) [31]. Technically, it was easy to count the axon varicosities of striatofugal neurons, as they were distinguishable from the thin axon fibers owing to their larger size (≥ 2 times the size of the axon fibers). This allowed us to measure the potential impact of individual striatal neurons on the GP, EP, SNr, and substantia nigra pars compacta (SNc). This study compared the relative number of axon varicosities in the GP between striosome and matrix direct pathway neurons. The results showed that 59%, 34%, and 7% of axon varicosities of matrix neurons were distributed in the SNr, GP, and EP, respectively. Meanwhile, 36%, 33%, 16%, and 15% of striosome neurons were found in the SNc, SNr, GP, and EP, respectively (Fig. 1). Direct pathway neurons have been reported to project to the GP in primates. Lévesque and Parent performed microiontophoretic injections of biotinylated dextran amine (BDA) and demonstrated that 24 of 27 reconstructed axons arborized into the three main striatal targets: GP, EP, and SNr [32]. These findings, obtained from different types of tracers, such as a Sindbis virus vector with membrane-targeting signals and BDA, and from different species of rodents and primates, align with previous reports. They suggest that the main targets of matrix direct pathway neurons are not exclusively SNr, but also the GP, rather than the EP [31]. Nadjar and colleagues' study with the retrograde tracer, cholera toxin subunit B, in sixteen female cynomolgus monkeys also support that the GP is directly innervated by direct pathway neurons [33]. Chan et al. used Adora2aCre and Drd1aCre mice with Cre-inducible mRuby2-T2A-Synaptophysin-eGFP adeno-associated virus (AAV). They demonstrated that the relative number of boutons in the GP formed by indirect pathway neurons was seven times greater than that formed by direct pathway neurons [34].

In a study by Cazorla et al., AAV-DIO-ChR2 was injected into the dorsal striatum of Drd1a-GFP and Drd2-GFP BAC transgenic mice; the findings revealed that not only the Drd2-expressing indirect pathway neurons but also the Drd1-expressing direct striatal neurons inhibited GP neurons. After laser stimulation of direct pathway striatal neurons of *Drd1a*-CRE mice, there was an inhibition in the basal firing rate of GP neurons compared with their prestimulation values (55%): however, the degree of inhibition was smaller than that in *Drd2*-CRE mice (90%) [35]. This indicates that even if there are fewer axon terminals in the axon collaterals of the direct pathway neurons than that in the indirect pathway neurons, the impact on the GP cannot be ignored. Their study also shows that the axon collaterals of the direct pathway neurons appear to be dynamically altered and inversely related to the excitability of indirect pathway neurons [35].

Our morphological study, utilizing AAV vectors, revealed that indirect pathway neurons exhibited a more extensive and denser axon distribution than direct pathway neurons [36]. Given that the density peaks of axons from both the direct and indirect pathway neurons nearly overlapped, it suggests the possibility of a cooperative interaction between both pathways within the GP [36].

#### Two types of GP neurons

In the classical model, the GP is regarded as a relay nucleus of the indirect pathway of the basal ganglia. Within this framework, the ‘prototypic’ GP neuron is a fast-firing GABAergic cell that supports a uniform function by innervating the STN and SNr [37–41]. This is despite reports of GP cellular heterogeneity [37, 42–51]. Single-cell labeling studies, which include microinjection studies and juxta-cellular labeling, have identified an unrecognized type of GP neurons. Research involving primates has indicated that 15–20% of pallidal axons project only to the striatum, as revealed by a study using BDA tracer [52].

Recently, pallidostriatal neurons, known as ‘arkypallidal’ neurons, were discovered through electrophysiological characterization and juxta-cellular labeling in a rat model of Parkinson’s disease [53]. The molecular expression patterns of prototypic and arkypallidal neurons, along with their innervation patterns and physiological features in healthy rats, have also been reported [50, 51]. Our study utilized a palGFP-expressing Sindbis viral vector to conduct single neuron tracing experiments. Our results indicated that the number of boutons in striatal collaterals of single prototypic neurons (excluding one prototypic neuron lacking striatal collaterals) was 332.40 ± 314.11 (mean ± SD) [54] (Fig. 1); this finding is consistent with the results of previous research employing neurobiotin for single neuron tracing studies, wherein the number of boutons in the striatal collaterals of single prototypic neurons was found to range from 478 to 1353 (mean 790.6 ± 404.2) [38, 55]. Even for the prototypic neurons, the average rates of the varicosity counts in the striatum, STN, and EP/SN, representing the total numbers of varicosities at all targeted sites, were 30.6%, 16.7%, and 52.7%, respectively [54]. Moreover, the absolute mean number of striatal axon varicosities from arkypallidal neurons was 736.75 ± 483.09 [54] (Fig. 1). Molecular profiles revealed a dichotomous cell classification. This classification is based on the finding that prototypic neurons expressing PV/Nkx2-1/Lhx6 are a significant population of STN-innervating GP neurons. Additionally, the striatum-innervating arkypallidal neurons in rats express FoxP2 [51]. It is known that arkypallidal neurons contribute toward 25% of the GP neuronal population.

The distinct electrophysiological properties exhibited by different projection types have been demonstrated in various studies. In vivo recordings demonstrated that prototypic neurons exhibited regular firing at a higher frequency than arkypallidal neurons in both anesthetized [51] and awake rats [56], as well as in mice [50]. Furthermore, the low-frequency firing of arkypallidal neurons was strongly coupled to the active phase of cortical oscillations in anesthetized rats [51]. Ex vivo recordings of brain slices from transgenic mice have demonstrated a dichotomy in the firing patterns of prototypical and arkypallidal neurons. Specifically, PV neurons fired more rapidly, while neuronal PAS domain protein 1 (Npas 1) neurons fired more slowly [57]. Additionally, the activity of arkypallidal neurons is consistent with animal behavior, as they are active during spontaneous movements in mice [50], whereas the activity of prototypic neurons is more diverse. Arkypallidal neurons are more strongly activated when rats are forced to stop their planned movements [56]. In vivo whole-cell recordings and optogenetics have revealed distinct electrophysiological and functional disparities between prototypic and arkypallidal neurons. These differences may be linked to the varied integrations observed among striatal neurons [58]. Moreover, a recent electrophysiological study, conducted both in vivo and in slices, showed that the irregular firing pattern of GP neurons largely relies on a local inhibitory synaptic barrage, which is caused by the spontaneous firing of other GP neurons [59]. More recently, electrophysiological experiments in awake monkeys also revealed two distinct types of GP neurons in the primates. The firing activities during tasks were different between these neuron types [60]. In this study, the authors carefully discussed that their results did not show the existence of arkypallidal neurons, since they determined neither molecular expression nor axonal projection. Although further research is requested to elucidate homology of neural circuitries between the rodents and primates, their study offer the possibility and necessity of consideration in the cell type-dependency of a basal ganglia circuitry in the primates.

#### How do direct pathway neurons affect GP neurons?

Cazorla et al. (2014) demonstrated that direct striatal neurons inhibit GP neurons in a heterogeneous and/or partial manner, whereas the inhibition from striatal indirect neurons is more intense and homogeneous. Consequently, identifying the specific types of GP neurons affected by the direct pathway axon collaterals is crucial [35]. The projection of axon collaterals from direct pathway neurons to GP neurons appears to be dependent on the neurotransmitters involved.

SP is a neuropeptide released by the striatal direct neurons [61–64], and its receptor is the neurokinin-1 receptor (NK-1R) [61, 65]. Available evidence supports the notion that SP is released by the axon collaterals of striatal direct pathway neurons and is received by NK-1R-expressing GP neurons. Our immunohistochemical study highlighted that the GP comprises two distinct types of NK-1R neurons: (i) a small population of robust immunopositive NK-1R neurons co-expressing choline acetyl transferase (ChAT) and (ii) a larger population of weakly immunopositive NK-1R neurons co-expressing PV and/or Lim-homeobox 6 (Lhx6) but not exhibiting forkhead box protein P2 (FoxP2) immunoreactivity [66]. Our retrograde tracing experiments demonstrated that the prototypic neurons expressed NK-1R with Lhx6 and/or PV, but not FoxP2. Furthermore, electrophysiological analysis showed a segregation of arkypallidal and prototypic neurons based on active and passive membrane properties. Neurons that displayed an inward current induced by an SP agonist were classified as prototypic neurons. Bath application of an NK-1R antagonist abolished this response. Collectively, these findings demonstrate that SP-responsive neurons comprise a part of the populations of prototypic neurons, as evidenced by both anatomical and electrophysiological data [66].

Conversely, a Rabies virus-mediated tracing study highlighted a preferential input–output organization whereby PV neurons and Npas1 neurons were preferentially connected by indirect and direct pathway neurons, respectively [34]. Furthermore, an optogenetic study revealed that the strength of the impact of direct pathway neurons originating from the dorsal striatum was lower on PV neurons than on Npas1 neurons [34]. While this review focuses on direct pathway neurons, Chan and his colleagues also made valuable discoveries about the relationship between indirect pathway neurons and the GP [34]. The combination of in vivo optical and chemogenetic tools with deep learning approaches in mice revealed that direct pathway neurons projecting to the SNr transmit synchronous motor-related information to the Npas1 neurons in the GP via axon collaterals. This suggests a model in which the axon collaterals of direct pathway neurons work in harmony with the canonical terminals in the SNr to support motor control by inhibiting Npas1 neurons in healthy mice [67].

Given the existence of specialized presynaptic terminals that facilitate the targeting of different neurotransmitters to specific outputs [68–71], it is possible that the direct pathway neurons inhibit arkypallidal neurons via GABA, while exciting prototypic neurons via SP. This possibility for the separable release of different neurotransmitters may introduce additional complexity to the basal ganglia network.

As previously mentioned, there have been reports of direct pathway neurons projecting to the GP in primates [32] and of the existence of the GP neurons that solely project to the striatum in primates [52]. However, there seems to be no report specifying which type of neurons the direct pathway neurons project to within the GP. Therefore, this section is based solely on studies conducted on rodents.

## Conclusions and perspectives for pathological state

What happens when direct pathway neurons innervate GP neurons in healthy and pathological states? If the direct pathway neurons excite prototypic neurons via SP in healthy animals, this shows that the direct pathway neurons lead to disinhibition of the EP/SNr via the striatum-GP-(STN)-EP/SNr pathway. This is followed by the inhibition of the thalamus and cerebral cortex, immediately after their disinhibition of the thalamus and cerebral cortex via the striatum-EP/SNr pathway. The ability of a direct pathway neuron to apply its own "brake" immediately after the "accelerator" may contribute to increasing the temporal resolution of the movement. Conversely, if the direct pathway neurons inhibit the arkypallidal neurons via GABA, they may act as a recurrent circuit between the GP and the striatum via the arkypallidal neurons. Given that Npas1 neurons and PV neurons are known to have opposing effects on motor control [72, 73], the inhibition of Npas1 neurons by GABA and excitation of PV neurons by SP may ultimately lead to the same behavioral performance. Regardless of the neurotransmitters involved, the GP serves as a valuable nucleus where direct pathway neurons can intervene in the indirect pathway. It is possible that the direct pathway neurons initiate the movement with the “accelerator” and then apply the “brake” to the self-initiated movement, thereby increasing the temporal resolution of the movement, akin to a one-man timing-based attack. However, most of these studies have been conducted in rodents. Since recent single-nucleus RNA sequencing has shown that the RNA expression profile of each region of the basal ganglia differs between primates and rodents [74], these anatomical and electrophysiological experiments need to be carried out with high reproducibility in primates.

In the context of pathological states, it is recognized that the typical symptom of Parkinson's disease, “hypokinesia,” is caused by the loss of dopaminergic neurons in SNc in humans. This symptom is generally attributed to the overactivity of the indirect pathway relative to the direct pathway. In Parkinson's disease, however, there are many conditions that cannot be explained by the conventional dual-pathway model, such as tremors, rigidity, and paradoxical movement. To solve this problem, the transcriptional profiles of neurons in each region of the basal ganglia may hold significant importance.

In humans with Parkinson’s disease, the degenerative loss of dopamine neurons in the SNc progresses gradually with age. This slow progression may lead to alterations in the direct and indirect pathways in the basal ganglia (see review article [75]). Although it is difficult to mimic this slow progression in rodent models of Parkinson's disease, the depletion of striatal dopamine through unilateral lesions of the nigrostriatal dopamine pathway in rodents has provided valuable insights into the progression of human Parkinson’s disease. Chan et al. used a chronic 6-OHDA lesion model of Parkinson’s disease and showed that the inputs from direct pathway neurons to Npas1 GP neurons were selectively strengthened [34]. They also showed that inputs from direct pathway neurons in the dorsal striatum suppress movement, and this selective strength may explain the hypokinesia observed in Parkinson’s disease [34]. Recent studies have reported that the axon collaterals of direct pathway neurons exhibit plasticity, and manipulation of these collaterals can alter animal behavior (35, 76, 77). This finding offers hope that therapeutic manipulation of basal ganglia neural circuits could be possible. Furthermore, mouse studies have reported that cell-specific stimulation of the GP can restore motor function in a Parkinson's disease model, which may have implications for the future of deep brain stimulation [78, 79]. The subpopulations of striatal projection neurons are coded through a combination of genes and along gradients, rather than through innervation patterns (see review article [80]).

The discovery of new networks may indeed make the basal ganglia appear complex at first glance. However, elucidating the real basal ganglia circuit is important not only for understanding healthy conditions but also for approaching neurodegenerative diseases.

## Data Availability

Not applicable.

## Abbreviations

GP: Globus pallidus
D1R: Dopamine D1 receptor
D2R: Dopamine D2 receptor
SP: Substance P
SNr: Substantia nigra pars reticulate
EP: Entopeduncular nucleus
GPi: Homologue of the internal segment of globus pallidus
GPe: Homologue of the external segment of globus pallidus
STN: Subthalamic nucleus
SNc: Substantia nigra pars compacta
BDA: Biotin-dextran amine
NK-1R: Neurokinin-1 receptor
PV: Parvalbumin
Lhx6: Lim-homeobox 6
FoxP2: Forkhead box protein P2
